# A conceptual approach to a citizens’ observatory – supporting community-based environmental governance

**DOI:** 10.1186/1476-069X-13-107

**Published:** 2014-12-12

**Authors:** Hai-Ying Liu, Mike Kobernus, David Broday, Alena Bartonova

**Affiliations:** Norwegian Institute for Air Research (NILU), Instituttveien 18, 2027 Kjeller, Noway; Division of Environmental, Water and Agricultural Engineering, Faculty of Civil & Environmental Engineering, Technion, Israel Institute of Technology, Haifa, 3200003 Israel

**Keywords:** Citizens’ Observatory, Citizen science, Environmental governance, Environmental monitoring, Top-down and bottom-up approach, Public participation

## Abstract

In recent years there has been a trend to view the Citizens’ Observatory as an increasingly essential tool that provides an approach for better observing, understanding, protecting and enhancing our environment. However, there is no consensus on how to develop such a system, nor is there any agreement on what a Citizens’ Observatory is and what results it could produce. The increase in the prevalence of Citizens’ Observatories globally has been mirrored by an increase in the number of variables that are monitored, the number of monitoring locations and the types of participating citizens. This calls for a more integrated approach to handle the emerging complexities involved in this field, but before this can be achieved, it is essential to establish a common foundation for Citizens’ Observatories and their usage. There are many aspects to a Citizens’ Observatory. One view is that its essence is a process that involves environmental monitoring, information gathering, data management and analysis, assessment and reporting systems. Hence, it requires the development of novel monitoring technologies and of advanced data management strategies to capture, analyse and survey the data, thus facilitating their exploitation for policy and society. Practically, there are many challenges in implementing the Citizens’ Observatory approach, such as ensuring effective citizens’ participation, dealing with data privacy, accounting for ethical and security requirements, and taking into account data standards, quality and reliability. These concerns all need to be addressed in a concerted way to provide a stable, reliable and scalable Citizens’ Observatory programme. On the other hand, the Citizens’ Observatory approach carries the promise of increasing the public’s awareness to risks in their environment, which has a corollary economic value, and enhancing data acquisition at low or no cost. In this paper, we first propose a conceptual framework for a Citizens’ Observatory programme as a system that supports and promotes community-based environmental governance. Next, we discuss some of the challenges involved in developing this approach. This work seeks to initiate a debate and help defining what is the Citizens’ Observatory, its potential role in environmental governance, and its validity as a tool for environmental research.

**Electronic supplementary material:**

The online version of this article (doi:10.1186/1476-069X-13-107) contains supplementary material, which is available to authorized users.

## Background

The word “environment” is derived from the old French, ‘environ’ which means encircle (en viron = in circle) or surround [1]. For us, the environment is literally the immediate surroundings within a circumference, with citizens at its centre. As such, this is a citizen and sensor centric perspective, and requires that we enable citizens to observe with their own senses and with sensors what is happening within their immediate circumference. This observational circle follows each individual as he navigates in his surroundings.

Recently, it has been increasingly suggested that the key to protecting our environment is to engage the average citizens, not only highly active environmentalists. Although our political, economic and administrative structures are designed to tackle our environmental concerns via large-scale policies and strategic decisions, these often leave citizens as unengaged and silent observers [2]. A major drawback of the traditional approaches to environmental monitoring (e.g., Earth Observation through satellites and in-situ observations through monitoring networks) is the sparsely collected data and their inherent remoteness from the citizens’ experience of the environment [3]. It is no longer sufficient to develop and provide passive lists of environmental indices or reports and inform citizens about changes in their environment. There is a need to engage citizens to find out how they can inform the community, and to empower citizens to improve their own health and wellbeing through actively making informed choices via the Citizens’ Observatory (CO) process [4–7]. Involving citizens at the local level by developing knowledge pools can help to create an atmosphere of active participation and generate a sustainable movement that can build over time [2]. Citizens have expectations to interact and participate in the decision making processes, and to be engaged in a dialogue about their communities, preferences and future. According to sociological research, the recent increase in pro-active participants of social IT media, with a particular focus on environmental issues, results from a shift from materialism to post-materialism [8] where more and more people are showing increasing interest in renewable and sustainable life style, which are the key objectives of the ‘Environmental Governance’ [9]. Developing a CO is a crucial step in bridging the gap between Environmental Governance and the public.

There is no a globally agreed and understood definition of Environmental Governance [10]. It can be interpreted in many different ways. In principle, Environmental Governance comprises the rules, practices, policies and institutions that shape how humans interact with the environment [11]. In this paper, ‘Environmental Governance’ refers to the processes of decision-making involved in the control and management of the environment for the purpose of attaining environmentally-sustainable development. Good Environmental Governance takes into account the role of all actors that impact the environment. From governments to Non-Governmental Organizations (NGOs), the private sector and civil society, the individual and the citizen groups, cooperation is critical to achieving effective governance that can help us move towards a more sustainable future [11]. A CO for supporting community-based environmental governance may be defined as the participation of citizens in monitoring the quality of the environment they live in, with the help of one or more of the following: (1) mobile devices of everyday utility; (2) specialized static and/or portable environmental and/or wearable health sensors, and (3) personal, subjective and/or objective observations, information, annotation and exchange routes, coming from social media technologies or other similar platforms [12]. In this context, the key aspect of Citizens’ Observatories (COs) is the direct involvement of ordinary citizens, and not just that of scientists/professionals in data collection as well as harnessing the citizens’ collective intelligence, i.e., the distributed information, experience and knowledge embodied within individuals and communities, to meet gaps that many areas of environmental management are still suffering from. Namely, CO should enable citizens’ participation in environmental monitoring, and contribute to environmental governance by providing relevant data and information that can help decision-makers make sound decisions. This can be advanced by providing citizens with a voice and supporting them with knowledge of their environment and as a consequence of raising their awareness.

The environmental concept of CO was first introduced in the project ‘Eye on Earth’ [13] with the European Environmental Agency (EEA) creating the first ‘official’ environmental portal that includes a CO on air, noise, nature, coral reefs and water quality. In addition, the EU has funded five CO-related projects under the FP7 topic ENV.2012.6.5-1 “Developing community-based environmental monitoring and information systems using innovative and novel earth observation applications” at the end of 2012, including (i) “Citclops – Citizens’ Observatory for coast and ocean optical monitoring”, 2012–2015 [14]; (ii) “Omniscientis – Odour monitoring and information system based on citizens and technology innovative sensors”, 2012–2014 [15]; (iii) “CITI-SENSE – Development of sensor-based Citizens’ Observatory Community for improving quality of life in cities”, 2012–2016 [16]; (iv) “WeSenseIt – Citizen Observatory of Water”, 2012–2016 [17]; and (v) “COBWEB – Citizen Observatory Web”, 2012–2016 [18]. On this basis it is expected that CO will have an increasing importance in supporting environmental governance and other applications over the next years.

As participants in a major EU FP7 project (CITI-SENSE) [16] as well as from work undertaken across several EU-funded projects, e.g., ENVIROFI, 2011–2013 [19] and HENVINET, 2007–2011 [20], which contained core components that were heavily based on the CO concept [21, 22], we have gained some insight on best practices for a CO programme. These have been implemented in the ongoing Citizens’ Observatory work in CITI-SENSE. The role of the Citizens’ Observatory in the project is to harmonise various independent/local Citizens’ Observatories and to develop coherent understanding of COs with regards to the overall project objectives.

While we agree that there might be various perspectives to each aspect of a CO, we have developed an initial concept that we believe can be applied to many CO initiatives in general. To this end, we propose a framework to support and influence community and policy priorities and associated decision making in environmental stewardship. Informed by existing approaches in CO-related environmental governance support, we propose a structural work system that enables effective citizens’ participation, data collection and interpretation, and information dissemination. Further, we review and discuss the main challenges faced by a CO programme in support of environmental governance. The aim of this paper is, therefore, to provide a platform for debate that should lead to a more comprehensive understanding of what a Citizens’ Observatory is, and how it can support environmental research and governance.

## Current citizens’ observatories

To put CO into perspective as an instrument to support community-based environmental decision making, it is useful to get a sense of the variety of COs within environmental media and relevant aspects [23]. A wealth of CO-related initiatives (e.g., Citizen Science, Community-Based Monitoring (CBM), Volunteered Geographic Information (VGI), Volunteered Environmental Monitoring (VEM), etc.) can be found around the globe. The Waterkeeper Alliance, for example, which includes the Riverkeeper, Lakekeeper, Baykeeper, and Coastkeeper programmes, which works towards the goals of ecosystem and water quality protection and enhancement has over 200 programmes in 15 nations [23–25]. The majority of these are located in the USA, Australia, India, Canada, and Russia [26–29]. A review of the academic- and non-academic-based literature indicates that these nations are among those leading many CBM initiatives and that by all indications the movement of ‘Citizen Science’ is increasing [21]. Kerr et al. (1994) [30] indicated a near tripling of new monitoring programmes with citizens’ engagement between 1988 and 1992, all related to water monitoring. Pretty (2003) [31] reported that since the 1990s, up to 500,000 new local population groups were established in varying environmental and social contexts. A review in 2006 showed that the increase of CBM has been particularly dramatic in the USA and Canada [32]. The cause for this rise has been attributed to an increase in public knowledge and concern about anthropogenic impacts on natural ecosystems [33–35] and recent public and NGOs concern about governmental monitoring of the environment and ecosystems [36]. In addition to COs involving the general public, private individuals and NGOs play many roles [33–36], the critical role of various institutions for observing earth and conserving the environment [16, 17, 19, 37], for achieving citizen participation in environmental monitoring [16, 17, 19, 38], for facilitating science-policy dialogue [20, 39], and for setting up Citizen Science as a discipline in its own right [40, 41], etc., is increasing as well [42].

In Europe, several ongoing national and international community-based environmental monitoring programmes currently exist, e.g., the EEA project ‘Eye on earth’ [13, 43, 44], the European Mobile and Mobility Industries Alliance (EMMIA) project Citi-Sense-MOB [45, 46], and the EU FP7 funded five CO-related projects (WeSenseIt [17], Omniscientis [15], COBWEB [18], Citclops [14], and CITI-SENSE [16]. According to Wiggins and Crowston (2011) [47], the recent decades have seen a growing emphasis on ‘scientifically sound practices and measurable goals of public education’. Some of the well-known projects were and are focused on nature and biodiversity, for example, The Open Air Laboratories [OPAL, [48]], The Big Butterfly Count [49], and Citizens’ Network for the Observation of Marine Biodiversity [COMBER, [50]]. However, there are many more CO-related programmes, encompassing different models of Citizen Science and within the environmental sciences these span a diverse range of subject (e.g., biodiversity, water, air, climate change, agriculture, disaster, etc.). To promote debate on the CO definition, concept and practices, we provide a brief review of nine programmes (Additional file 1): Citclops, CITI-SENSE, Citi-Sense-MOB, COBWEB, Eye on Earth, Omniscientis, Waterkeeper Alliance, WeSenseIt, The Big Butterfly Count. We focus on (i) the aim/purpose of each programme; (ii) its geographic scope; (iii) project duration; (iv) target groups; (v) monitoring parameters; (vi) data collection and interpretation, visualization and information dissemination technologies. These six properties determine the potential of the programmes for supporting informed decision-making. These programmes can be classified into:

- International programmes whose objectives are to develop Citizens’ Observatories using innovative earth observation technologies (air, water, odour, biodiversity, etc.), e.g., CITI-SENSE, WeSenseIt, COBWEB, Citclops, Omniscientis.
- International programmes whose objectives focus on enabling greater access to and sharing of environmental and societal data, e.g., Eye on Earth.
- National and/or international programmes whose objectives are on creating community-based environmental monitoring in varying environmental and social contexts towards the goal of ecosystem, biodiversity and environmental quality protection, e.g., the Waterkeeper Alliance programmes, The Big Butterfly Count, Citi-Sense-MOB.

From the information in Additional file 1, we have identified the following characteristics that seem to be vital for the Citizens’ Observatories: (i) A CO should involve citizens as active partners in environmental monitoring and decision-making, since this is central for protecting and enhancing our environment; (ii) CO-related environmental monitoring should target an array of natural resources and/or a range of environmental components; (iii) Generally, the involvement of citizens in CO has multiple purposes, with education and raising public awareness being the most common objectives associated with a CO [45, 51–53]; (iv) There is value in CO as a way to bring community groups together. CO, like other forms of civic engagement, can build social capital within the community [53, 54]; (v) Evaluation of the effectiveness of a CO as well as of the public involvement in environmental decision-making is generally lacking. There are a number of questions about its potential as a democratizing force in environmental policy and management [52, 53, 55]. However, given the many contending conceptions within democratic theory (e.g., direct, representative, participatory, minimal, deliberative, aggregative, etc.) [56], it should be noted that this aspect is a complex subject with no “one size fits all”. Nevertheless, a CO in the environmental domain has shown its potential role to address issues of environmental equity and to improve social justice [57].

## Conceptual framework of citizens’ observatory in support of environmental governance

### Definition of citizens’ observatory

There is no clear definition of CO available yet. In the broadest sense, a CO for supporting community-based environmental governance may be defined as ‘the citizens’ own observations and understanding of environmentally-related problems, and in particularly as reporting and commenting on them’. As such, the CO promotes communicates and supports sharing of technological solutions (e.g., sensors, mobile apps, web portals) and community participatory governance methods (e.g., aided by various social media streams) among citizens. A CO is also open and democratic, enabling the possibility for anyone who is interested or willing to contribute and participate in earth observation and environmental conservation [58, 59]. It also promote a more active role for the community with regards to understanding the environment, since citizens are traditionally considered to be merely consumers of information services at the very end of the information chain [4] and not as data providers. This definition reveals three core components that underpin some of its objectives, i.e., raising the citizens’ environmental awareness; enabling dialogue among citizens, scientists and policy/decision makers and supporting data exchange among citizens, scientists and other stakeholders.

### Citizens’ observatory in support of environmental governance

We believe that the above three components of CO can explain the major links between Citizens’ Observatory and environmental governance, and in fact as the three pillars that sustain a Citizens’ Observatory for supporting environmental governance. In the context of Citizens’ Observatories contribution to environmental governance, it is important to recognise that citizens are not monolithic, with CO stakeholders/user groups [59] including individual or groups of volunteers, scientists, government authorities, emergency services, etc. Hence, various stakeholder actors in a CO have different behaviours, intentions, interrelations, agendas, interests, as well as influence, resources and power on decision-making and political processes [60, 61].

### Raising awareness

Information is available to us in a myriad of ways and from many sources: newsprint, radio, television, online portals and mobile device. In fact, there is so much information that it is sometimes hard to keep track of what we need, or even to really understand what we need to know. Recently awareness grew that “it is no longer sufficient to develop passive lists or report to ‘inform’ citizens of changes in our environment. We need to engage with citizens and ask how they can ‘inform’ us” (Prof. Jacqueline McGlade, Executive Director, European Environment Agency) [2, 62]. At the recent 2013 Green Week conference, the European Environment commissioner, Janez Potočnik, reinforced this when he stated that “We have learned that public awareness is of key importance for the implementation of existing air policy, as well as for the success of any future air pollution strategy” [63]. Clearly, getting the useful ‘message’ across to the public, in the right way, and thereby effectively raising public awareness, is critical. The first criterion therefore is to determine who we would like to get the message to, and to target those users in a way that ensures a certain level of interest.

In previous projects (e.g., ACCENT, 2009–2011 [64], HENVINET [20], and ENVIROFI [19]) we have attempted to engage users through various campaigns, including mass emailing, printed media such as brochures, online video presentations and workshops in the Café Scientifique format. These methods generated a sufficiently moderate number of public users interested in knowing more about the project but ultimately did not create a self-sustaining community of users that are willing to engage/participate for a long period in a community forum that is based on social network platform(s). Hence, while it could be argued that we were moderately successful, it was clear that we did not really create a viable, sustainable community. What was missing was the emphasis on knowledge transfer. Raising awareness is not just about alerting the public or recruiting users; it is just as much about helping those users understand the problems and concerns so that they can make informed decisions of their own. While these platforms did include expert users who could answer questions about relevant environmental issues, this does not automatically translate into true knowledge transfer. An additional factor is to ensure that the communities’ opinions, thoughts, questions, etc., are not only heard, but are valued. For this, we need to provide a platform that support a dialogue among the users in a CO programme.

Furthermore, to facilitate citizens play an active role in the data collection process (e.g., via portable sensors and smart phones, information and communication technology), as well as harness the citizens’ collective intelligence (e.g., using apps and social medias), a self-sustaining community of users should be formed, which exchange data/information and knowledge, reach the expert who could answer questions about relevant environmental issues, and disseminate information to understand environmental issues. The Chinese Proverb “tell me and I’ll forget; show me and I may remember; involve me and I’ll understand”, does apply in this context.

### Enabling dialogues

Successful multi-stakeholder dialogues are critical to ensuring a deeper level of interest of the stakeholders, especially the general public. At the most basic level, these can take the form of peer-to-peer as well as public-to-expert. Yet, any discussion forum needs to have a comprehensive and consistently active membership drawn from a multidisciplinary volunteer ‘workforce’. Nothing kills a communication portal quicker than low levels of active participation. Only if regular activity of a varied group of users is achieved one can likely see a sustained growth over time, as more people begin to participate than fall away. It cannot be overemphasized that this is not a place for passive participation and that static information portals guarantee a quick demise. Social media applications can be employed as a platform for initiating dialogue but in itself, this is ultimately insufficient. It is critical to move to a more advanced level, since multi-stakeholder dialogues are more than just question and answer or discussion forum style communication. They must include technology based information gathering and exchange systems, including sensors, smart-phones, personal subjective observations, etc. These will create a much broader canvas for information gathering and for data exchange.

### Data exchange

Data exchange is much more than just pushing data to users, and it goes beyond the sharing of ideas or questions. In a Citizens’ Observatory context, this must include a variety of Volunteered Geographical Information (VGI) observation types, in addition to personnel observations on an array of topics, such as physical wellbeing, perceived environmental effects and even just personal opinions. The key for this is user that is encouraged to provide data inputs regularly, and finds value in the way that this information is used. The user’s peers should also find value in these data and be further encouraged to make their own observations available. An important aspect is that all data, not just electronic sensor data, has a geo-temporal marker.

Public users are now in a position to use micro-sensors in increasing numbers due to advances in technology and lowering costs. An individual might purchase sensors for different reasons and tie them to a network that collect, store and disseminate data. Such platforms become increasingly possible. However, while this results in more data being generated it does not necessarily engage the users, who might be entirely passive data providers. For example, pollen data is generally very limited, so major generalizations are often made about the prevalence of pollen in any given area. If individuals reported the presence of particular types of pollen in a specific area, this could be of great interest to others who also have an allergic reaction to that particular pollen. Therefore engaging users in providing personal observations on their perception of the environment can have beneficial consequences for others, which will further encourage others to participate and share their own observations. Finally, presenting information that combines heterogeneous data sources which includes VGI data allows the stakeholders, in particular public users, to see how their individual contributions add to the value chain, ultimately creating a reinforcing mechanism that will help to create a self-sustaining community.

### Citizens’ observatory framework

**Figure 1:**
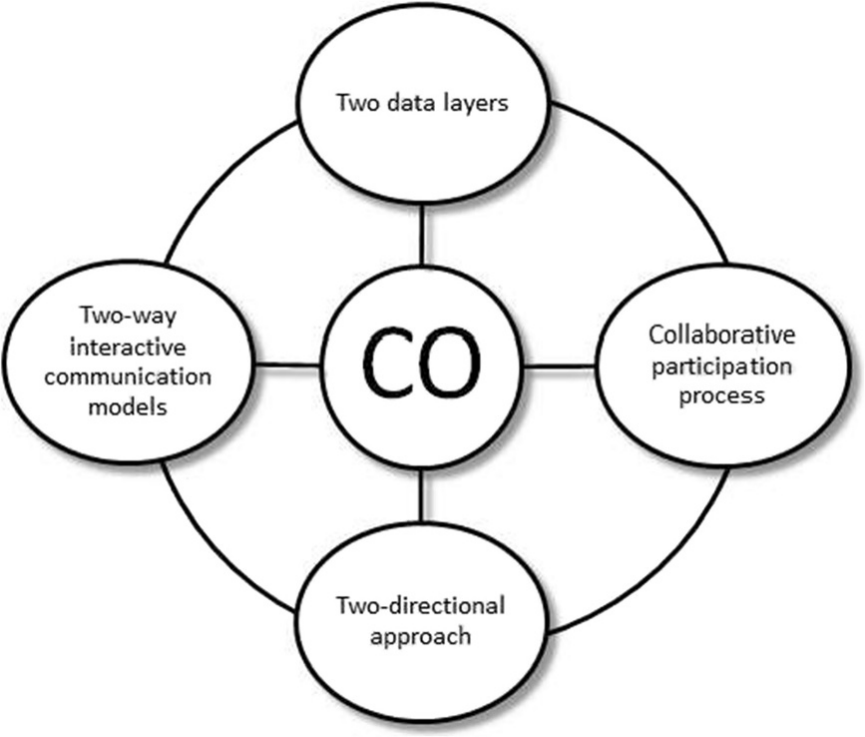
**Conceptual frameworks to a Citizens’ Observatory.** Grouped as follows: (i) Collaborative participation process; (ii) Two data layers: hard layer and soft layer; (iii) Two-directional approach: top-down and bottom-up, and (iv) Two-way interactive communication models.

Based upon the definition of the CO we have given and our understanding of how a Citizens’ Observatory may supports environmental governance, we propose that a Citizen’s Observatory comprises four aspects, which we refer to as the CO framework (Figure 1), as follows: (i) Collaborative participation process; (ii) Two data layers: hard layer comprising data generated from sensors and the soft layer comprising data generated from citizens; (iii) Two-directional approach: top-down and bottom-up and (iv) Two-way interactive communication model.

In the following sections we elaborate each of these concepts in turn.

#### Collaborative participation process

Citizen participation should be considered throughout the entire chain of monitoring-data-assessment-reporting within an environmental monitoring programme. Key to this is ensuring that citizens are both motivated and equipped to influence the decision making process. This approach will also enable and motivate the citizens to change their personal behaviour and priorities in order to improve their environments. For example, indoor air quality in schools can be improved considerably if all children and staff take off their shoes before entering the classroom. This change in behaviour directly affects the environment and is possible due to the active participation of the citizens.

Moving away from the traditional one-way transfer of knowledge between scientists and citizens is important. Collaborative participation demands that the citizens not only consume information, but also provide it, leading to the joint production of knowledge (where multiple forms of expertise, for example from researchers, practitioners and the public) are valued equally in the production of knowledge [65]. Fernandez-Gimenez et al. [66] have posited that a collaborative participation process in environmental monitoring can lead to shared environmental understanding among diverse participants, build trust internally and credibility externally, foster social learning and community-building, and advance adaptive management [66].

#### Two data layers

Many citizens and volunteers are equipped today with Global Positioning System (GPS) enabled devices, digital cameras and numerous other resources, turning citizens into potential resources for creating and sharing publicly relevant information [66]. In other words, citizens become part of the information chain as data suppliers, not just consumers. In addition to collecting existing data and information from relevant programmes and projects, the two data layer approach can be applied in a CO programme, utilizing citizens as mobile sensors as well as implementing physical sensors. The hard layer (physical sensor layer) includes static and portable devices for sensing and transferring environmental information via mobile devices. The objective here is to have a large number of sensors providing spatial patterns and temporal evolution of the changing environment and real-time information for decision-making. For example, in several current projects (e.g., CITI-SENSE, Citi-Sense-MOB), static sensors will be installed in parallel with existing stationary monitoring networks, while portable sensors will be carried by citizens [16, 45]. The soft layer (Human layer) is harnessing citizens’ own observations of their surroundings/environment. This includes social trends and social activity, online participation in public forums (such as Facebook page, Twitter account, LinkedIn group, etc.), participative Geotagging and sharing of their own subjective/objective observations on their perceived environment.

#### Top-down and bottom-up approaches

Top-down and bottom-up are strategies of information processing and knowledge ordering, mostly involving software, but also other humanistic and scientific theories [67]. In many cases top-down is used as a synonym of analysis or decomposition, and bottom-up of synthesis [68]. Both top-down and bottom-up approaches exist in CO-related programmes (e.g., CITI-SENSE, Citi-Sense-MOB). To meet the challenges in the data coverage, a combined top-down and bottom-up approach is often used [68]. Top-down approaches are typically research-led (expert) and often start with the formulation of visions of future direction. At the same time, a broad variety of bottom-up initiatives are taken by different public groups (citizens) who develop and try out new approaches to meet the challenges as they see them. Most of these initiatives are not guided by broad future visions and focus on specific aspects [69, 70]. Accordingly, Citizens’ Observatories can be seen as a combination of top-down and a multi-layer bottom-up approach. This can be defined and interpreted differently. The method proposed here, involves a combined top-down and bottom-up approach in a CO programme and can interpret the use of citizen science as a two-way data connection between the researchers and citizen scientists who work from opposite approaches (Top-down and bottom-up) [71, 72]. The current scientific knowledge and policy analysis (top-down gathered knowledge) are combined with local knowledge, experiences and perceptions (bottom-up collected information through ordinary citizens’ observatories). This approach creates a platform for exchange of information in two directions, and an involvement and engagement with all stakeholders which is crucial if a sustainable CO programme is to take place.

**Figure 2:**
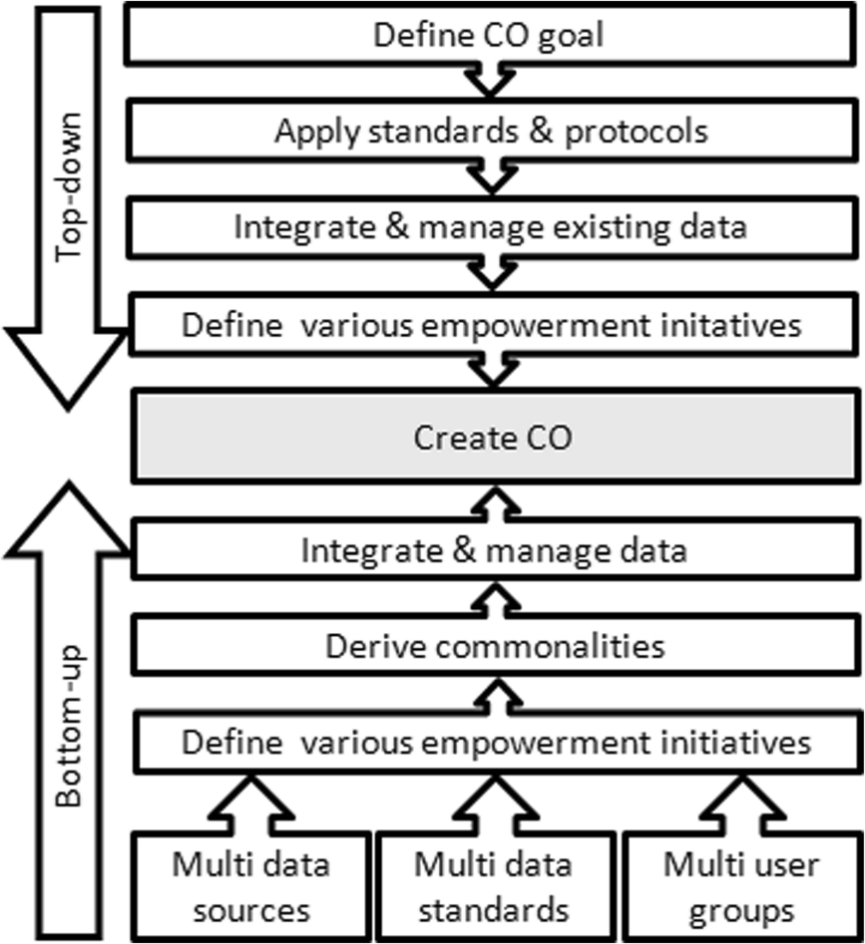
**A top-down and bottom-up approach have the same goals via different paths, but both allow for individual case study data analysis and integrated data analysis.**

For multiple location-based case studies in a CO programme (e.g., CITI-SENSE [16, 73]), the combined top-done and bottom-up approach can be defined by the following: (i) The key components of the top-down CO approach includes the definition of the COs goals, selecting and applying the necessary standards, protocols, sampling designs and methodologies, wherever these have one identical purpose in various case studies within one environmental domain. (ii) Crucial to the multiple layer bottom-up COs approach are heterogeneous data sources that can accommodate multiple data standards, conflicting requirements from diverse user groups and a definite need to develop methodologies that are able to integrate diverse systems and ultimately synthesize data of many types and formats. Together, these two approaches aim to minimize the differences, and maximize what is similar, among multiple systems, enabling both individual case study data analysis and integrated data analysis to be performed [21]. Figure 2 presents the top-down and bottom-up process in this context. The top-down approach is to ensure individual case studies within a CO programme have coordinated data types and acquisition, so that they can be analysed in an integrated manner at a later stage. The bottom-up approach enables the integration and synthesizing of results from individual case studies, these results arising from multiple and potentially conflicting needs.

In addition, the top-down approach may be considered to be management driven, and the bottom-up approach may be considered as driven by the needs of the client/s. The ultimate goal is that both approaches should be merged. It is important to understand that these are not separate approaches and that they should be run in parallel, not individually.

#### Two-way interactive communication model

In order to harness environmental data and knowledge to effectively and efficiently management of environmental issues, a CO which will enable citizens and communities to take on a new role in the environmental management chain needs to be developed: a shift from the traditional one-way communication paradigm in which citizens are passive information receivers [74], into a two-way communication model, in which citizens become active stakeholders in information capturing, evaluation and communication [4]. As a result, citizens will become important in two ways: (i) as data providers through the direct involvement of user communities in the data provision and collection process; and (ii) by solving consensus tasks, e.g., by collecting multiple assessments from citizens [75] and gathering information on the environment.

To realize this, there is the need for understanding the citizens’ demographics [76] to develop a CO platform that meets their needs. Beyond demographics, community needs should be defined by the community stakeholders themselves through efforts such as strategic planning, community visioning, design charrettes, etc. [77, 78]. Furthermore, technical capacity need to be built as well for facilitating citizens observing environment, collecting and exchanging data, communicating and visualizing observing results back to the broader community.

## Structural work system of citizens’ observatory

**Figure 3:**
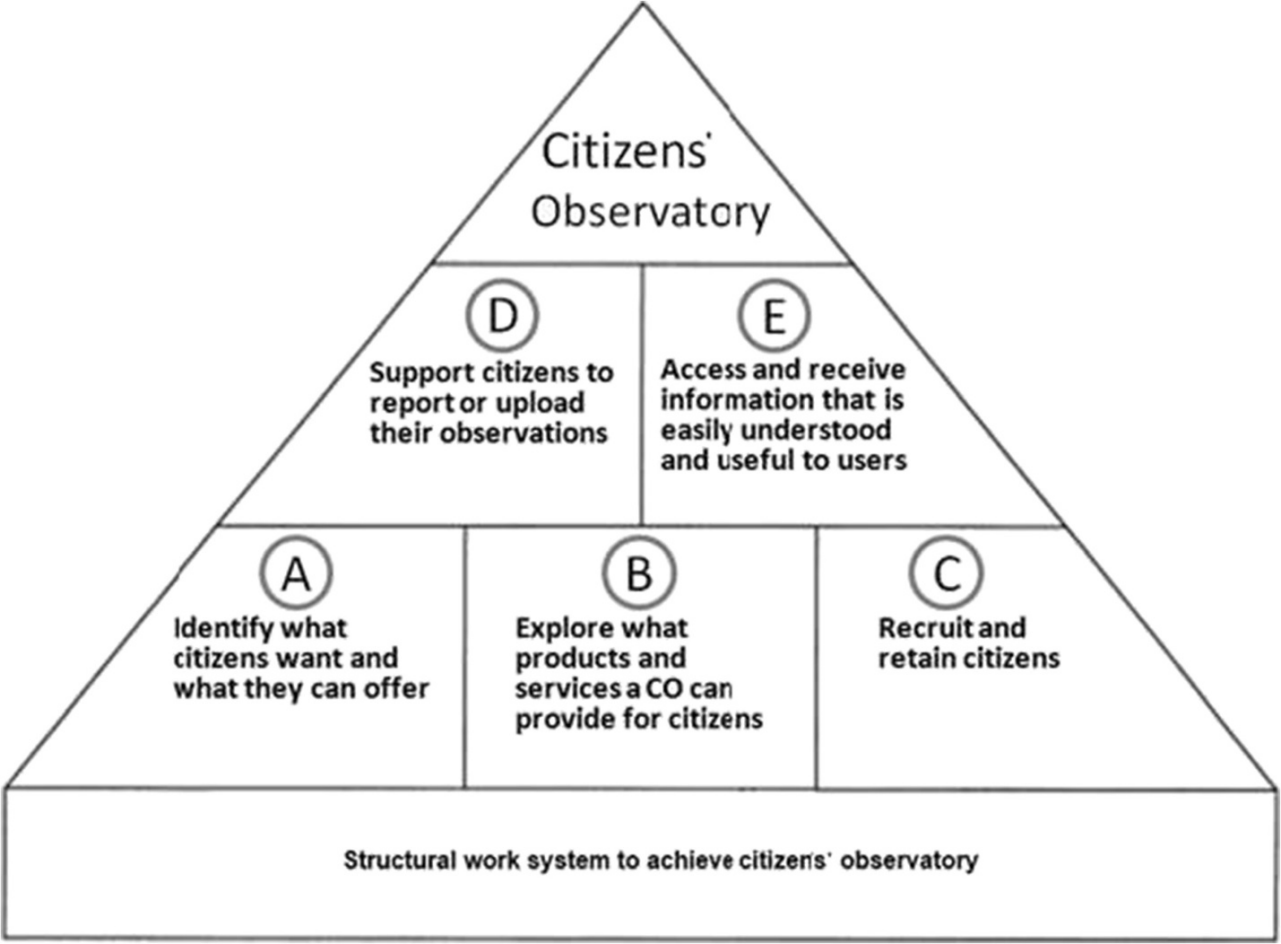
**Sequential aspects of a Citizens’ Observatory programme.**

To establish a CO and to make it useful to society, we need to collaborate with citizens, citizens groups and their representatives, to identify their needs and concerns, and with the representatives of the local municipality or environmental protection office, to identify their interests and needs. This information can then be cast into a SWOT (strengths, weaknesses, opportunities and threats) analysis approach [79] to promote the dialogue between all stakeholders. The review of the varieties and characteristics of different ongoing Citizens’ Observatories that focus on environmental issues reveals a set of five sequential aspects that underlie the CO skeleton and support effective citizens’ participation (Figure 3): (A) Citizens’ participation in identifying what a CO can offer to provide information and knowledge in response to public concerns. This is achieved mainly by a dialogue among the stakeholders; (B) Citizens’ participation in exploration of what products and services a CO can provide for the citizens. This involves systematizing and structuring citizens-created content to make it appealing for use by citizens during their normal daily life; (C) Recruitment and retaining of citizens to participate in and contribute to environmental governance: further clarify the purpose, scope and expected impact of the CO, identify motivations that will promote citizens to contribute to and take part in the CO, and encourage public participation in data collection and interpretation; (D) Obtaining public participation in the relevant decision making and/or in changing their related personal priorities and behaviour by gaining access to environmental data, knowledge and experience, and by using tools that can support citizens to report or upload their objective/subjective observations; (E) Providing tools to access and receive timely information on relevant environmental issues in a manner that is both easily understood and useful to the users.

## Challenges and development needs

### Challenges

The essence of a Citizens’ Observatory lies in public widespread engagement in collecting data that can be used in environmental decision-making, which is relevant to public concerns. Although the CO concept is becoming a more common practice for environmental management, scepticism still exists about the quality of the data collected as well as its usefulness for environmental policy [52]. Furthermore, it has been suggested [80] that improved citizens’ monitoring can even have adverse effects on environmental quality. This dichotomy suggests that the following areas should receive careful consideration: (i) Data quality (i.e., accuracy and uncertainty) – especially when comparing crowd-sourced and reference data; (ii) Data privacy and security – sharing of data and information requires strong ethical and security considerations; (iii) Data interpretation – qualitative indicators such as “quality of life”, “wellbeing”, “happiness”, etc., should be developed in parallel with more quantitative indicators that are based not only on individual perception, but on an integrated sensor network; (iv) Systematisation and structuring of citizens-created content and feedback – establishing a viable model(s) to support decisions and empower the public [81]; (v) Involving and maintaining a broad spectrum of society – implementing various location-specific and target group-tailored tools in recruiting and sustaining citizens’ participation in environmental monitoring [82].

Whereas we recognize the critical importance of the above issues, which must be solved to ensure the viability of the CO model, we believe that the value it carries and its pluralistic and democratic foundations override many of the reservations currently still associated with it.

### Development needs

In terms of ensuring a usable CO, some key challenges that we have faced during the course of several projects where we developed a CO component include the following: (i) We need to adequately promote the CO platform and tools, to raising awareness, recruiting and sustaining citizens’ participation. The old adage, “if you build it, they will come”, does not apply. (ii) We need a good understanding of citizens’ demographics in order to develop the CO platform to meet their needs, especially as they change; (iii) We need to build a long lasting infrastructure that uses open standards, is easily exploitable through an open Application Programming Interface (API), can be widely accessed, extended and maintained, and is seen as a generic environmental enabler rather than a project specific outcome; (iv) We need to address and evaluate Citizens’ Voice and Accountability (CV&A) in the social and political context in which Citizens’ Observatories are embedded [82–84], to actively promote the CV&A concepts as important dimensions of good environmental governance [84], to address CO’s potential role to influence environmental equity and to improve social justice [75]; and (v) we need to develop particular channels and mechanisms that can underpin the sound environmental-social-political actions in which Citizens’ Observatories are addressed, in a manner which facilitates citizens to influence environmental governing priorities and processes.

Surveying technology evolves quickly but issues relating to data collection and analysis always prevail. The latter include: (i) Building needed technical capacity and overcoming the ‘digital divide’ for environmental monitoring, data exchange, visualizing and communicating results back to the broader users [83, 85]; (ii) Managing and analysing increasing data volumes, variety and velocity [86]; (iii) Reducing measurement uncertainties; (iv) Developing reliable and fast quality assurance/quality control (QA/QC) tools that can work in real-time; and (v) Increasing need for interdisciplinary use of data, integration of different types of data. Whereas solutions toward many of these potential limiting factors already exist, progress in computational tools that can process large volumes of data and enable analysis of large volume of data is foreseen [87]. Decision support methods and tools (e.g., Aguila [88]; the Numerical Unit Spread Assessment and Pedigree (NUSAP) system [89, 90]) can be used to deal, to some extent, with the inherent data uncertainty. Expert elicitation can be used to deal with some aspect of uncertainty by consulting experts as a means to derive preliminary estimates for information [91–95]. And more traditional methods can be used to overcome the ‘digital divide’, by making data available in other methods, such as web, or even TV and Radio (e.g., high pollen warnings, dust, etc.).

Furthermore, data privacy issues need to be addressed in a spatial-temporal data mining context. In the Geographic Privacy-Aware Knowledge Discovery and Delivery (GeoPKDD) project, Giannotti and Pedreschi (2010) [96] investigated various scientific and technological issues of mobility data, open problems, and roadmap. They found that privacy issues related to Information Communication Technologies (ICT) can only be addressed through an alliance of technology, legal regulations and social norms. In the meanwhile, increasingly sophisticated privacy-preserving data mining techniques are being studied and need to be further developed. The final aim is to achieve appropriate levels of anonymity by means of controlled transformation of data and/or patterns but with limited distortion, to avoid the undesired side effects on privacy, to preserve the possibility of discovering useful patterns and trends.

In addition, an issue with data quality and its use in shaping environmental policy is the gap between science and policy [95, 96] caused by poor timing, ambiguous results and lack of relevant data [97]. Addressing these concerns requires approaches that are both interdisciplinary and engages scientists with societal needs, developmental needs and the implementation of a variety of novel methods and tools to bridge the communication gap [98]. In this regard, we believe Citizens’ Observatories provide the possibility by addressing several of the concerns mentioned, such as increased spatial resolution, up-to-the-minute data coverage and improved environmental awareness leading to a stronger public voice.

## Conclusions

In this paper, we lay the groundwork for a debate on the conceptual framework for developing a Citizens’ Observatory. Based upon the review of different ongoing COs and of CO-related programmes in the environmental domain, we have identified key elements and qualities which are essential for a CO programme: (i) Be a unique virtual place to gather and share data from a variety of sources: novel sensor-technologies, open environmental data from public and national sources, and personal perceptions and textual/graphical contribution; and (ii) Extract and make use of relevant citizens-related data and provide multimodal services for citizens, communities and authorities.

Based upon our experience in Citizens’ Observatory from the CITI-SENSE and Citi-Sense-MOB projects, we posit that citizens observing and understanding environment related problems, as well as reporting and commenting on them within a dedicated platform, is the key to a successful CO implementation.

To better understand the links between Citizens’ Observatory and environmental governance, we first propose three pillars: (i) Raising awareness; (ii) Enabling dialogue; and (iii) Data exchange. In addition, we suggest a CO framework which provides: (i) A collaborative participation process; (ii) Two data layers: a hard layer and a soft layer; (iii) Two-directional approach: top-down and bottom-up; and (iv) A two-way interactive communication model. With these processes in place, citizens will be in a position to participate actively in environmental surveillance in a way that will benefit them in a timely manner.

Current CO programmes attempt demonstrate the main aspects needed to effectively address citizens’ participation. These include participation in data collection, data interpretation and information delivery. Alternatively, this can be expressed as: A) Identifying what citizens want and what citizens can offer; B) Exploring what products and services a CO can provide for the citizens; C) Recruiting and retaining citizens to participate in and contribute to environmental governance; D) Providing tools that support citizens to report their observations, inference and concerns; and E) Supplying tools to access/receive timely information on the environment in a manner that is both easily understood and useful.

We believe that achieving these milestones will facilitate the CO objective of engaging citizens and stakeholders in participating in environmental surveillance. This, in turn, will contribute to better informed decisions, contribute to improved quality of life, and ensure that the interest of people in the environment and its impact on human health and wellbeing continues to grow. By raising a debate on this topic we hope to further the understanding and potential of Citizens’ Observatory and their wider acceptance in environmental monitoring.

## Electronic supplementary material

Additional file 1: **Overview of nine Citizens’ Observatories programmes with their aim, location, period, target groups, monitoring parameter(s), data collection, and communication and visualization methodologies.** For acronyms please see the text. (DOCX 20 KB)

## Abbreviations

CBM: Community-Based Monitoring
Citclops: Citizens’ observatory for coast and ocean optical monitoring
CITI-SENSE: Development of sensor-based Citizens’ Observatory Community for improving quality of life in cities
Citi-Sense-MOB: Mobile services for environment and health citizen’s observatory
CO: Citizens’ observatory
CO_2_: Carbon dioxide
COs: Citizens’ observatories
COBWEB: Citizen observatory web
Copernicus: Global monitoring for environment and security
CVA: Citizens’ Voice and Accountability
DALYs: The disability-adjusted life years
EEA: European environmental agency
EMMIA: The European mobile and mobility industries alliance
ENVIROFI: The environmental observation web and its service applications within the future internet
GeoPKDD: Geographic Privacy-aware knowledge discovery and delivery
GEOSSS: Global earth observation system of systems
GO: The global observatory
GPS: Global Positioning System
HENVINET: Health and environmental network systems
ICT: Information communication technology
NUSAP: Numerical unit spread assessment and pedigree
Omniscientis: Odour monitoring and information system based on citizen and technology innovative sensors
QA/QC: Quality assurance/quality control
SDI: Spatial data infrastructure
SEIS: Shared Environmental Information System
VEM: Volunteered Environmental Monitoring
VGI: Volunteered geographical information
SWOT: Strengths, weaknesses, opportunities and threats
WeSenseIt: Citizen water observatories
WNBR: The world network of biosphere reserves.
