# Supplementary material for: A conceptual approach to a citizens’ observatory – supporting community-based environmental governance
Source: Environ Health. 2014 Dec 12;13:107. doi: 10.1186/1476-069X-13-107 (PMC4320485; doi:10.1186/1476-069X-13-107)
Supplement: Supplementary file 1 — Additional file 1: Overview of nine Citizens’ Observatories programmes with their aim, location, period, target groups, monitoring parameter(s), data collection, and communication and visualization methodologies. For acronyms please see the text. (DOCX 20 KB) [file 12940_2014_825_MOESM1_ESM.docx]

## Additional file 1

| **Project acronym** | **Aim/purpose** | **Location** | **Period** | **Target groups** | **Monitoring parameter(s)** | **Data collection, communication and visualization** |
| --- | --- | --- | --- | --- | --- | --- |
| **Citclops** | To develop an observatory based on citizens’ science applications for the bio-optical monitoring of coast and ocean. | Spain-wide: more than 200 control spots located at 200 different beaches, Spain | 2012-2015 | Citizens (individuals and associations); Environmentally engaged persons; Yachting-club members; Scuba divers; Students; Local administrations | Coast and ocean bio-optical monitoring, including colour, transparency and fluorescence of seawater | Data collection: Low-cost sensors combined with people acting as data carriers, contextual information (e.g., geo-referencing) and a community-based Internet platform, taking into account existing experiences (e.g., Secchi Dip-In, Coastwatch Europe and Oil Reporter)  Data communication: Online (Bluetooth and project web portal) and offline  Data visualization: GIS data integration; Data interpretation; Knowledge based integration; Interpreting collected data through artificial intelligence techniques. |
| **CITI-SENSE** | To empower citizens to participate in environmental governance, by developing up to 30 Citizens’ Observatories supporting services related to outdoor air quality, indoor school air quality and environmental perception in public spaces of societal concern. | Europe: Barcelona, Spain; Belgrade, Serbia; Edinburgh, United Kingdom; Haifa, Israel; Ljubljana, Slovenia; Oslo, Norway; Ostrava, Czech Republic; Vienna, Austria; Vitoria, Spain | 2012-2016 | General public; Health interest groups; Local government(s); School students, staff, students’ parents, school governors; Researchers; Enterprises | Urban air quality, indoor school air quality, environmental perception in public spaces and their effects on wellbeing/comfort | Data collection: Traditional monitoring stations; Small, low-cost sensors (both static and portable); Air quality models, Citizens’ volunteered geographical information through mobile and web apps; Global Navigation Satellite System  Data communication: Online (GPRS and/or Bluetooth, project web portal), and offline  Data visualization: web and mobile applications |
| **Citi-Sense-MOB** | To develop, test and demonstrate mobile services that exhibit the potential for mobile technologies in the environmental health and climate change domain, using the concept of a citizens’ observatory. | Norway: Oslo, Norway | 2013-2015 | General public; Health interest groups; Local government(s); Researchers; Enterprises | Urban air quality and its effects on wellbeing/comfort | Data collection: Traditional monitoring stations; Small, low-cost sensors (both static and portable); Air quality models, Citizens’ volunteered geographical information through Mobile and web apps; Global Navigation Satellite System  Data communication: Online (GPRS and/or Bluetooth, project web portal), and offline  Data visualization: web and mobile applications |
| **COBWEB** | To create a test bed environment which will enable citizens living within Biosphere Reserves to collect environmental data using mobile devices. | Europe: Dyfi BR, Wales; the Wadden Sea and Hallig Islands, Germany; the Gorge of Samaria and Mt. Olympus, Greece | 2012-2016 | Citizens; Public authorities; Researchers; Decision-makers; Policy-makers; Commercial companies; Local communities | Biosphere reserve including biological monitoring, flooding, creation and validation of earth observation data products | Data collection: Crowd sourced environmental data; Sensors in the environment; Authoritative data; Data generated from groups on earth observations  Data communication: open e-collaboration framework  Data visualization: Validated, quality approved and compliant data available through GEOSS. |

## Additional file 1 (*Cont*.).

| **Project acronym** | **Aim/purpose** | **Location** | **Period** | **Target groups** | **Monitoring parameter(s)** | **Data collection, communication and visualization** |
| --- | --- | --- | --- | --- | --- | --- |
| **Eye on Earth** | To allow users to visualise, create, interact with, and share maps and data on air pollution, water quality and noise levels. | Global | 2010-Current | Citizens; Scientists and academics; Environmental agencies; NGOs | Water, air, noise, climate change, biodiversity, land use | Data collection: Crowd source’ environmental information on earth  Data communication: Online (project web portal)  Data visualization: GIS-based web maps |
| **Omniscientis** | To combine the active participation of the stakeholders, especially citizens, with the implementation of innovative technologies to improve the governance of odour nuisance. | Europe: A pig farm, Austria; A major industrial site, Belgium | 2012-2014 | Citizens; Experts; Environmental NGOs; Local authorities; Industries | Odour and its effects on human health including odour perception, discomfort and nuisance from industries | Data collection: In-situ sensors (e-noses and meteorological station); Citizens’ observatories via a geo-mobile application; Specific odour dispersion model  Data communication: Web-based service platform and offline  Data visualization: web and mobile applications |
| **The Big Butterfly Count** | To raise awareness of the importance of butterflies and moths, to encourage sustainable agriculture and helping to protect the environment. | UK-wide: parks, school grounds, gardens, fields or forest | 2010-Current | Public; Butterfly Conservation; UK government | The numbers of butterfly, species of butterfly | Data collection:  Data communication: Online via project’s website or via a smartphone app  Data visualization: Butterfly identification chart, butterfly pictures |
| **Waterkeeper Alliance** | To connect and support local waterkeeper programs to provide a voice for waterways and their communities worldwide. | Global: over 200 Waterkeeper Organizations on six continents | 1999- | Citizens and government(s) | Water quality | Data collection: Satellite images and mapping; Aerial reconnaissance and photography; On-the-water observation and sampling.  Data communication: both online and offline  Data visualization: layer-based  Data visualization |
| **WeSenseIt** | To enable citizens to become active stakeholders in information capturing, evaluation and communication for the water environment including flood risk. | Europe: Doncaster, United Kingdom; Delftland, Netherlands; Alto Adriatico, Italy | 2012-2016 | Citizens; Authorities; Academic institutions; Research centres; Industries | Water environment including flood risk (e.g., sonar water  Level, temperature, humidity, wind speed, rainfall, soil moisture, water  depth gauges, water velocity, snow depth, water quality) | Data collection: Innovative sensor devices developed within the project used by the citizens; Citizens’ collective intelligence via social media (e.g. Twitter, Facebook, etc.); Communities to upload key information to the observatory via mobile app.  Data communication: Web-based service platform  Data visualization: web and mobile applications |
